# Responding to Food Industry Initiatives to Be "Part of the Solution": Comment on "‘Part of the Solution’: Food Corporation Strategies for Regulatory Capture and Legitimacy"

**DOI:** 10.34172/ijhpm.2022.7241

**Published:** 2022-06-28

**Authors:** Nicholas Freudenberg

**Affiliations:** CUNY Graduate School of Public Health and Health Policy, City University of New York, New York City, NY, USA.

**Keywords:** Food Policy, Food Industry, Commercial Determinants

## Abstract

In response to growing concerns about chronic diseases, food insecurity, low-wage food labor, and global warming, the food industry has developed new strategies to respond to its critics and pursue its business and political goals. As Lacy-Nicholas and Williams described in a recent review, the food industry has expanded its repertoire from opposition to critics to appeasement, co-option, and partnerships.^1^ Defining themselves as "part of the solution," the food industry seeks to disarm its opponents, shift policy debates to favor its interests, or delay decisions that jeopardize its profits or power. This commentary explores how health professionals, can respond to this changing repertoire. Lessons from previous campaigns to control harmful industry practices,^2^ suggest that no single strategy will counter changing food industry efforts to achieve its goals. Thus, advocates must consider a portfolio of approaches that can be deployed in response to changing circumstances, industry tactics, and threats to health.

 Ipropose five strategies to respond to changing practices of the food industry deployed to resist efforts to modify its health-harming practices. These include activities to:

1. De-normalize health-damaging food industry practices,
2. Build cross-cutting alliances to challenge food industry power,
3. Reduce monopoly concentration in food sector,
4. Define principles for public health, nutrition, and food organizations to interact with food industry, and
5. Promote ideas that build a vision for alternatives to current corporate food regimes.

## Possible Solutions

###  1. De-normalize Health-Damaging Food Industry Practices

A key contribution to more effective regulation of the tobacco industry was successful efforts to de-normalize practices such as advertising to children and young people, making false and misleading claims to the public, and lying to government officials. For tobacco control, de-normalization was defined as activities that “shift the focus from individual smokers’ judgment to corporate misbehavior showing how the industry has ‘operated outside the boundaries of civilized corporate behavior’ by marketing a deadly product.”^3^

While the tobacco and food industries differ in important ways, the two use common practices to advance their private interests at the expense of the public good. By making evidence-based public health, political, economic, and moral arguments that show how food industry marketing, product formulation, worker protection, and environmental practices violate well-accepted values of protecting children’s health, speaking truthfully to the public, and respecting democratic values, public health advocates can shift social norms and eventually the law in ways that better support health.^4^ Examples of such campaigns include youth-led unhealthy food countermarketing campaigns, Mexico’s sugar tax, and London’s ban of advertising unhealthy food on its public transport system. Box 1 lists some food industry practices that could serve as targets for de-normalization campaigns.

**Box 1. Possible Food Industry Practices for Targeted De-normalization Campaigns^8^**

1. Marketing unhealthy foods to children, low-income, or racial/ethnic populations with higher risks of dietary diseases.
2. Providing low wages, limited benefits, and unsafe working conditions to essential food workers.
3. Advertising foods by making false health claims.
4. Marketing and selling food products banned in high income countries in low- and middle-income countries.
5. Employing agricultural and production practices that lead to carbon and greenhouse gas emissions.
6. Using food production practices that are toxic to human or planetary health.
7. Avoiding or evading taxes.
8. Lobbying or making campaign contributions to weaken consumer, public health or environmental protections.

Social media, rapidly emerging as a key platform for marketing unhealthy food, offers health advocates new opportunities for de-normalization. Consumer-facing corporations such as McDonalds, PepsiCo, and Coca Cola value their public reputations. Corporate Accountability International’s campaign to retire Ronald McDonald, for example, challenged the company’s advertising designed to subvert parental control of their young children’s diet.^5^ Similarly, evidence that Nestlé’s total food portfolio contained more unhealthy than healthy products undercut its desired global image as a healthy brand.^6^ This illustrates how social and other media can be used to undermine food corporations’ “credibility engineering” designed to show they care about children’s health.^7^

It should be noted that the practices described in Box 1 are employed, albeit in differing patterns, across all sectors of the food industry including agricultural producers, fast food chains, beverage makers, and supermarket chains.

###  2. Build Cross-cutting Alliances to Challenge Food Industry Power

 Over the last century, public health campaigns that engage various constituencies in challenging corporate power at the local, national, or global levels are more likely to succeed than those supported by any single interest group. Certainly, the emerging food industry strategies that Lacy-Nichols and Williams describe depend on mobilizing support from multiple influential stakeholders.

The food industry’s success in using its “soft power”^9^ to achieve its goals results from use of its power, wealth, and political savvy to win over elected officials, government agencies, media representatives, and sectors of the public. To challenge these efforts, proponents of healthier food systems will need to build alliances that can mobilize countervailing power and identify windows of opportunity to exercise this power to achieve meaningful reforms. Two examples are the Coalition For Immokalee Workers successful campaign to increase wages and safety protections for tomato pickers in Florida^4^ and International Baby Food Action Network boycott of Nestlé designed to change the infant formula industry’s unethical marketing practices.^10^ In these cases, a coalition of diverse constituencies was able to challenge industry power on several fronts, wining at least partial victories that also set the stage for future campaigns. These alliances can also help to link individuals seeking to change in their personal behavior – giving up meat or cooking more at home—to the political campaigns that can change the programs and policies that often make healthier choices more difficult than unhealthy ones.

###  3. Reduce Monopoly Concentration in Food Sector 

In the last decade, monopoly concentration of the food industry has increased significantly so that three to five corporations now dominate each sector of the industry.^11^ In food and other sectors, monopoly concentration harms health by increasing the power of industry actor and reducing the influence of competitors, government, farmers, civil society groups, and consumers. Monopoly concentration shapes health by raising prices, limiting competition to better meet consumer needs, increasing industry resources for marketing, lobbying, and campaign contributions, and reducing the power of government to protect public health.

 Monopoly concentration also contributes to precarious globalized supply chains, created by transnational corporations to maximize profits but not human welfare. The coronavirus disease 2019 (COVID-19) pandemic and Russia’s invasion of the Ukraine disrupted global food trade, exacerbated food insecurity—and generated windfall corporate profits. These problems that could have been reduced by global food policies that valued national food sovereignty, limited profits during global crises, and discouraged concentration of grain, meat, and other food producers.

 By strengthening and enforcing anti-trust laws, adequately funding regulatory agencies, imposing higher taxes on windfall profits won through monopoly consolidation, and urging international agencies such as Food and Agriculture Organization, World Bank, and International Monetary Fund to adopt policies that discourage monopoly concentration, food advocates can begin to level the playing fields in which monopolies and their critics vie to shape policy.

###  4. Define Ground Rules for Public Health, Nutrition, and Food Organizations to Interact With Food Industry 

As food industry actors develop new ways to appease or coopt their critics,^1^ public health actors need to decide their terms of engagement with industry. In setting the rules for partnerships with the food industry, the goals of advocates should not be some unattainable moral purity but rather a confidence that these interactions lead to meaningful improvements in food justice, defined as “the ways that race, class, gender, and other forms of inequality affect …food systems”^4^ (p. 5).

 Lessons from reducing inappropriate influences in other industries may be helpful. Section 5.3 of the Framework Convention for Tobacco Control specifies that while public officials can talk to tobacco industry representatives, these actors must be excluded from actual policy deliberations, based on the premise they have an irreconcilable conflict of interest between private profit and public good. Another approach is to condition discussions with industry representatives on a prior commitment from commercial actors not to lobby, secretly fund, or make campaign contributions to groups opposing stronger public health regulation of the industry. A third approach is to create fully independent bodies to evaluate and report on the success of corporate public health partnerships.

In some cases, making a corporation and its allies the target for advocacy, rather than a partner in dialogue, may be more effective in achieving the desired result. Targeting the corporate and institutional opponents of their proposed reforms, argue Young and Schwartz, “can undermine their adversaries’ ability or commitment to oppose the changes.”^12^ By challenging the safety or healthfulness of a company’s products, services, or practices, advocates can make it easier for government officials and politicians to support their reforms.

###  5. Promote Ideas That Build a Vision for Alternative to Current Corporate Food Regime 

 Food (and other) industry leaders recognize that victories in the policy arena depend on promoting and winning acceptance for ideas that support their business and political goals. Among the beliefs that food industry representatives who claim to be “part of the solution” advance are that: (1) individuals are responsible for their own food choices, (2) freedom of choice is the highest value for consumers, (3) market solutions are always preferable to government solutions, and (4) the food industry knows best which solutions are practical or impractical. Through their public relations campaigns, advertising, corporate philanthropy, lobbying, and political contributions, they promote these ideas and support other actors who endorse them. Proponents of the current corporate global food regime use these ideas to build support for the status quo and discourage alternatives.

To create a coherent, appealing alterative to this market ideology, food and nutrition advocates should develop, debate, and disseminate both challenges to its assumptions and a vision of a food regime that better supports health, the environment, and sustainable development. Two ideas endorsed by the tobacco control movement — the right to breathe clean air trumps the right to smoke and communities have the right to protect their children and young people from messages that endanger their health — illustrate how such ideas can set the stage for political victories. Box 2 lists ideas that if widely accepted could facilitate reforms in food policy.

**Box 2.Promoting Ideas That Build Support for Alternatives to Corporate Food Regimes**

** Ideas That Challenge Food Industry Messages**

1. While the corporate food regime offers the benefits of convenience and low costs to many, its cumulative costs to human and planetary health, social justice, equity, and economic prosperity are too high.^13^
2. Ask parents, “who would you rather trust to look out for your children’s well-being: Coca Cola and McDonalds or health professionals?” The real threat comes not from the “nanny state” but from corporate nannies who intrude on parental rights to exploit children’s vulnerabilities to grow their profits.
3. When most choices the food industry offers its consumers are unhealthy ones and when these options are promoted more vigorously than healthier ones, freedom of choice is an illusion.

** Ideas That Support an Alternative Vision for Food Systems**

1. The food industry claims there is no alternative to the food system it has created. But around the world, families, communities, and nations are creating alternatives such as community-based food systems, public food procurement, worker and consumer cooperatives, public markets, and others, proving in practice that other approaches are possible.
2. In many places around the world, the public sector contributes substantially to the food system including through agricultural and food subsidies, public procurement, and food benefit programs. By insisting that public money support public, not private goals, the public food sector can create a foundation for food system change.^14^
3. While the globalized food industry has immense power and wealth, it is neither monolithic nor homogeneous. Finding ways to divide industry actors may facilitate policy successes.

## Conclusion

The five strategies proposed here consider lessons from previous efforts to change the practices of food and other industries whose operations harm health and from insights into the changing repertoire of tactics the food industry deploys to counteract its critics, as described in emerging scholarship on the commercial determinants of health.^1,15^ By developing their own integrated, flexible comprehensive repertoire of public health responses to the strategies of the global food industry, advocates for a healthier, more equitable and sustainable global diet and food system can contribute to transforming this industry from a fundamental driver of ill health into a force for improved human and planetary health.

## Ethical issues

 Not applicable.

## Competing interests

 Author declares that he has no competing interests.

## Author’s contribution

 NF is the single author of the paper.
